# Adsorption of Macrolide Antibiotics and a Metabolite onto Polyethylene Terephthalate and Polyethylene Microplastics in Aquatic Environments

**DOI:** 10.3390/antibiotics13050408

**Published:** 2024-04-29

**Authors:** Carmen Mejías, Julia Martín, Laura Martín-Pozo, Juan Luis Santos, Irene Aparicio, Esteban Alonso

**Affiliations:** Departamento de Química Analítica, Escuela Politécnica Superior, Universidad de Sevilla, E-41011 Seville, Spain; cmpadilla@us.es (C.M.); lpozo@us.es (L.M.-P.); jlsantos@us.es (J.L.S.); iaparicio@us.es (I.A.); ealonso@us.es (E.A.)

**Keywords:** microplastics, antibiotics, polyethylene, polyethylene terephthalate, macrolides

## Abstract

Microplastics (MPs) and antibiotics are emerging pollutants widely found in aquatic environments, potentially causing environmental harm. MPs may act as carriers for antibiotics, affecting their environmental distribution. This study investigates the adsorption of four macrolide antibiotics and a metabolite onto two types of MPs: polyethylene terephthalate (PET) and polyethylene (PE). Results revealed a linear isotherm adsorption model, with higher adsorption to PET than to PE (*R*^2^ > 0.936 for PE and *R*^2^ > 0.910 for PET). Hydrophobic interactions and hydrogen bonding could be the main adsorption mechanisms, with pore filling potentially involved. Reduced particle size enhances adsorption due to the increase of active adsorption sites. This increasement is more pronounced in PE than in PET, leading to an 11.6% increase in the average adsorption of all macrolides to PE, compared to only 5.1% to PET. Dissolved organic matter inhibits adsorption (azithromycin adsorption to PE was reduced from 12% to 5.1%), while salinity enhances it just until 1% salinity. pH slightly influences adsorption, with maximal adsorption at neutral pH. Results in real samples showed that complexity of the matrix decreased adsorption. Overall, these findings indicate that PE and PET MPs can be a vector of macrolides in aquatic environments.

## 1. Introduction

Plastic has become an integral part of human life, finding applications in construction, building, agriculture, packaging, industry, electronics, and more [1]. The global production of plastic has increased enormously worldwide over the last sixty years, posing a significant threat to the environment [2]. Due to improper management, plastics end up entering the environment, where they can persist for a long period due to their high stability and resistance to degrading by microorganisms [3]. In recent years, particular attention has been given to microplastics (MPs), plastic particles smaller than 5 mm, due to their potential harmful impact on organisms and ecosystems [4]. MPs have been found in sludge [5], soil [6], seawater, wastewater [7,8], and even in living organisms [9]. In the environment, MPs of different natures have been described, with two of the most commonly found being polyethylene (PE) and polyethylene terephthalate (PET) owing to their widespread use [10]. The concern with MPs not only lies in their toxicity by themselves, but also, due to their small size and large surface area, in their ability to adsorb and concentrate organic pollutants or act as vectors, transferring harmful contaminants to organisms and promoting bioaccumulation [1,11,12,13]. For instance, Zhang et al. (2019) found out that polystyrene (PS) MPs could facilitate the bioaccumulation of roxithromycin (RXM) in red tilapia [14].

Antibiotics are emerging organic pollutants of special concern that can have negative effects on biota at different trophic levels and on human health [15,16,17,18]. The increasing global consumption of antibiotics has led to their widespread presence in the environment, promoting the development of antibiotic-resistant genes and enhancing microbial antibiotic resistance, among other effects [16,19]. Among them, macrolide antibiotics are extensively consumed, resulting in their release into the environment along with their metabolites. Consequently, this type of antibiotics and their metabolites are frequently detected in aquatic environments worldwide [20]. For example, among the reported concentrations of macrolides in the aquatic environment over the last two decades, the highest concentration was observed in the surface water of the Madrid region (Spain), reaching 3847 ng/L of erythromycin (ERY), which is a representative example antibiotic pollution in influent water of WWTPs [20,21].

Antibiotics represent one of the most problematic organic pollutants when associated with MPs. The adsorption of antibiotics onto MPs can have a significant influence on the occurrence of macrolides in the environment. MPs may affect their migration and act as vectors, allowing for transfer to different compartments, but also act as reservoirs, accumulating them over time [8,15,19,22,23,24]. For example, Wang et al. (2021) reported that the contribution of polyethylene (PE) to tetracycline antibiotic migration could be affected by the way they were exposed to environments [15]. Syranidou et al. (2022) showed that MPs could also serve as carriers of gene transfer, facilitating gene exchange between different bacteria and thereby propagating pathogenicity and antibiotic resistant in the environment [24].

Therefore, it is of utmost importance to study the adsorption behavior of antibiotic contaminants to MPs in the aquatic environment, as well as the influence of MPs’ characteristics and environmental factors such as salinity or the amount of dissolved organic matter, which have shown to have a significant impact. Regarding macrolide antibiotics, only two studies have been published, but they have focused only on one or two macrolide compounds, without considering the influence of MPs’ features or ambient conditions [17,25]. Moreover, to our knowledge, no study has previously reported the sorption behavior of macrolide metabolites to MPs. These studies, including metabolites or transformation products, are essential because they could exhibit toxicity similar to the parent compound but potentially different sorption behavior [26].

The aim of the present work was to evaluate the adsorption capacity of four macrolide antibiotics (RXM, ERY, azithromycin (AZM), and clarithromycin (CLM)) and a metabolite (N-desmethylclarithromycin, DM-CLM) on two types of microplastics commonly found in aquatic systems, PE and PET. To achieve this, a series of batch experiments were conducted to: (i) characterize and identify potential structural changes experienced by the microplastics after the adsorption process, using techniques such as scanning electron microscopy (SEM), Fourier transform infrared spectroscopy (FTIR) or X-ray diffraction (XRD); (ii) construct adsorption isotherms by applying representative models to define the adsorption behavior; (iii) investigate the effect of the physicochemical properties MPs particles and environmental factors on adsorption capacity; and iv) assess the adsorption in a real scenario using municipal water, river water, and wastewater.

## 2. Results and Discussion

### 2.1. Microplastics Characterization 

**Figure 1:**
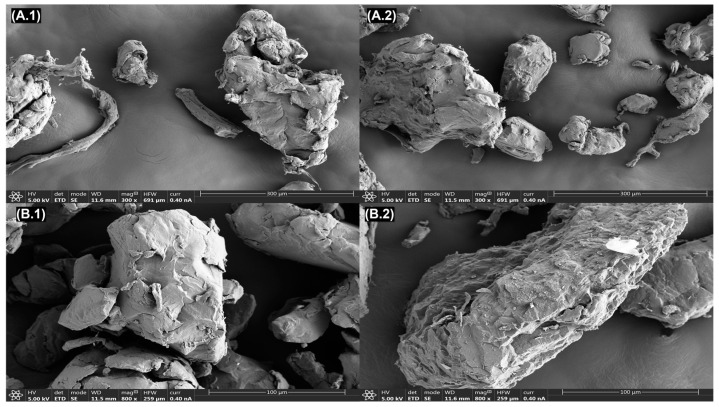
SEM images of PE before (**A.1**) and after (**A.2**) adsorption and of PET before (**B.1**) and after (**B.2**) the adsorption process.

MPs were characterized before and after the batch experiments, with the aim of identifying any material changes due to the adsorption process. Surface area, morphology, and degree of crystallinity of MPs were examined as these properties may play an essential role in the adsorption capacities of the antibiotics [22]. SEM was used to observe the surface morphology of MPs. Figure 1 shows SEM micrographs of PE and PET particles (300 µm size) prior and after the adsorption of macrolides under standard conditions. Both MPs were irregular pellet particles with a rough surface. The surface of PE particles was relatively coarse because of multiple irregular folds, creating voids and pore spaces [27]. The surface of PET was also rough, although slightly smoother, as there were fewer noticeable voids compared to PE. The surfaces of both MPs experienced a subtle increase in roughness after the adsorption process, as shown in the images; however, no significant drastic changes in morphology were observed.

Additionally, the surface area and particle sizes were assessed. These data can be found in Table S1. PE particles exhibited a lower surface area than PET pellets (0.0686 m^2^/g and 0.1345 m^2^/g, respectively), which was in concordance with their particle size (252 µm for PE and 119 µm for PET); as particle size decreases, surface area increases. 

No significant differences were observed in the average particle size during the adsorption procedure. Only a decrease of 3.8% in the size of PE particles and an increase of 5.4% in the size of PET particles was noted. In contrast, the surface area for both MPs increased after batch experiments, resulting in an estimated Brunauer–Emmett–Teller (BET) surface area of 0.0726 m^2^/g for PE and 0.1444 m^2^/g for PET. This gain in surface area could be attributed to the increased roughness of the pellets observed after adsorption as result of a potential wearing away of the particles. 

FTIR was performed to visualize potential new functional groups in the MPs surface after the adsorption process. FTIR transmittance typical spectra of PE can be seen in Figure S1: two bands are assignable to –CH_2_ asymmetric stretching (2919 and 2851 cm^−1^), one band is assignable to –CH_3_ symmetric deformation (1377 cm^−1^), another band in associated with rocking deformation (731–720 cm^−1^), and, finally, two bands are assignable to bending deformation (1473 and 1463 cm^−1^) [28]. The deficiency of functional groups and low surface energy of PE makes it almost chemically inert, which is confirmed by the stability of the FTIR peaks [29]. The same peaks were found in the FTIR transmittance spectra of PE after adsorption which indicates that no new functional groups were added to the MP structure, nor were new bonds formed. Differences in intensity of the peaks were observed, but no new peaks were created. The FTIR transmittance typical spectra of PET can be seen in Figure S2. Bands at 3100–2800 cm^−1^ have been attributed to aromatic and aliphatic –CH bond stretching, one band to the ester carbonyl bond stretching (1720 cm^−1^), one band to the ester group stretching (1300 cm^−1^) and, finally, one band to the –CH_2_ group (1100 cm^−1^) [30]. As in the case of PE, the only slight difference was the intensity of the diffraction peaks in the spectra prior and after adsorption, but no new peaks appeared. 

XRD analysis was used to determine the degree of crystallinity of MPs particles. Polymers with a high degree of crystallinity exhibit sharp diffraction peaks, while those with a substantial amorphous component have bowed diffraction peaks. The typical peak for PE is characterized by a sharp peak indicating that PE is a crystalline MP, while the typical peaks for PET are bun peaks, which correlates with an amorphous polymer (Figure S3). Organic contaminants can easily mobilize into the loosely arranged polymer chains of amorphous polymers but are constrained in crystalline glassy polymers due to their highly dense molecular structures [31]. The peak number and their shape after the adsorption of macrolides were observed to be similar as before adsorption, but the peak intensity was lower, which indicates that MPs become more amorphous after adsorption.

### 2.2. Adsorption Kinetics, Isotherms, and Adsorption Mechanism

The adsorption process of the macrolides by the studied MPs was investigated. To analyze the adsorption kinetics, pseudo-first and pseudo-second order adsorption kinetic models were applied to the obtained data. Results can be seen in Table S2 in Supplementary Materials. The pseudo-first order (PFO) model assumes that the physical diffusion process is the rate-limiting step, whereas the pseudo-second order (PSO) model relates the adsorption rate to the squares of the quantity of unoccupied adsorption sites on the surface, involving chemical interactions between antibiotics and MPs [32]. Parameters were better adjusted to a PSO model (*R*^2^ > 0.968), which has previously been reported as the most common model in MP adsorption [10,32,33]. This suggests that the adsorption of these antibiotics to PE and PET involves both physical and chemical adsorption processes, likely influenced by sorption onto surface sites, mass transfer, and intraparticle diffusion [32,33]. Consistently, prior research has found that the antibiotic adsorption to MPs fitted the PSO kinetic model [10]. Moreover, the *q_e_* values of macrolide adsorption (amount of compound adsorbed at the equilibrium) calculated from both the pseudo-first and pseudo-second order kinetics were relatively low and similar between models. However, values obtained were slightly higher for PET compared to PE, indicating that PET has a higher adsorption capacity. 

To study the adsorption isotherms of macrolides to MPs, Freundlich, Langmuir and Linear isotherm models were used to fit the equilibrium data. The Langmuir isotherm model assumes that the surface of the adsorbent is covered by a large number of active sites, where each site can be considered to be occupied by an adsorbed molecule. The sites are all equivalent, and it is believed that the adsorbed molecules do not interact with each other or jump from one site to another. Moreover, adsorption is complete when all the sites are occupied, corresponding to a monolayer of adsorbate. The Linear isotherm model corresponds to a homogeneous surface with infinite active sites, the adsorbed molecules do not interact with each other, and it states that adsorption is directly proportional to adsorbate concentration, and it applies only at low concentrations. However, because most surfaces are heterogeneous, there are multiple sites available for adsorption; i.e., the adsorption varies from one site to another, as described by the Freundlich isotherm model. Parameters derived from these models are summarized in Table 1. These results showed a better fit of the adsorption data to the Linear model than to the other two models, considering the higher regression coefficients (*R*^2^ > 0.936 for PE and *R*^2^ > 0.910 for PET) obtained with this Linear model. Similar results were obtained for ERY on PS particles [25], as well as for other pharmaceuticals on PE MPs [34]. These consistent findings indicate that adsorption occurred on the uniform surface through monolayer adsorption by partitioning. All 1/n values of the Freundlich model were <1.0, indicating that adsorption of macrolides to both MPs was a favorable and concentration-dependent process. *K*_d_ values were in the range from 0.002 to 0.008 L/g for PE and from 0.005 to 0.011 L/g for PET; however, similar sorption capacity was reached by different macrolides. Moreover, the *q*_max_ values ranged from 0.654 to 1.131 mg/g for PET and from 0.027 to 0.075 mg/g for PE, indicating that sorption follows the order PET > PE, which was consistent with the data from the adsorption kinetics. This could be attributed to the deficiency of functional groups of PE which make it almost chemically inert. 

**Figure 2:**
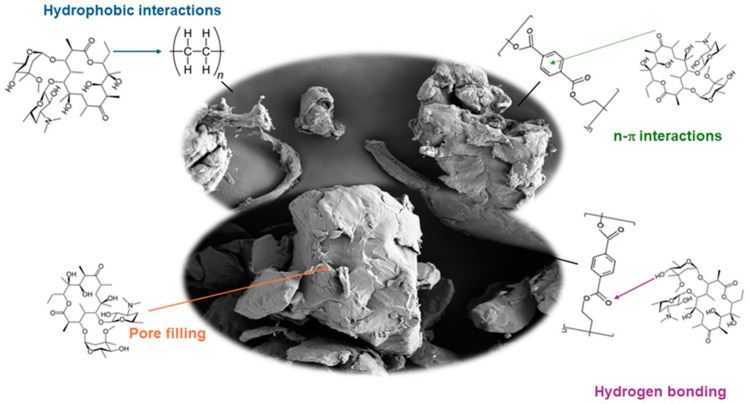
Adsorption mechanism.

An adsorption mechanism is used to describe the types of molecular interactions taking place during the adsorption process. In Figure 2, the main adsorption mechanisms between macrolides and MPs are represented. The main adsorption mechanisms between antibiotics and MPs previously reported are hydrophobic interactions, electrostatic interactions, partitioning, π–π bonding, pore filling, hydrogen bonding, and Van der Waals forces [3,11,12,15]. Adsorption mechanisms depend on antibiotics and MPs’ physicochemical characteristics [10]. The polarity of MPs is a key factor affecting adsorption capacity. According to their functional groups, PE is considered a non-polar MP while PET is considered a polar MP. Generally, those organic pollutants with a high log octanol–water partition coefficient (*K*_ow_) (>3) value tend to be absorbed by MPs more easily through hydrophobic interactions [22]. Macrolides considered in this study have log *K*_ow_ values ranging from 2.75 to 4.02 (Table S3), indicating that they may be susceptible to adsorption via hydrophobic interactions with non-polar MPs. Consequently, considering the hydrophobic nature of PE, such interactions could be one of the sorption mechanisms. In addition, the polar characteristics of PET may facilitate interaction with pollutants via hydrogen bonds (between ester groups present in PET structure and hydroxyl functional groups present in macrolides structure). n–π interactions between the aromatic ring of PET and ketone group of macrolides could be also involved. These results are confirmed by FTIR and XRD results because they showed that only physisorption occurred. As no new covalent peaks appeared in the characterization, no new bond was formed, so chemisorption is not involved. However, pore filling might also be implied in the sorption process in both cases [24], as was observed in results obtained by SEM. PET MPs contains a benzene ring, so it is an MP capable of forming π–π bonds; however, macrolides do not have any benzene rings, so this adsorption mechanism must be discarded. The influence of electrostatic interactions is discussed later in the study of pH (Section 2.4).

### 2.3. Influence of Dosage and Size of Microplastics

**Figure 3:**
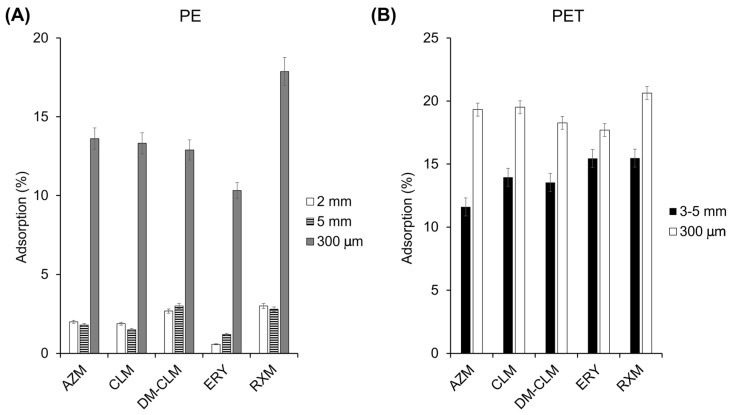
Adsorption of macrolides on PE (**A**) and PET (**B**) with different particles sizes.

The dosage and particle size of MPs can exert an influence in the adsorption. First, possible differences in the adsorption capacity of antibiotic macrolides to PE and PET were evaluated using a similar size. Second, different amounts of MPs and different particle sizes were assessed (Figure 3). According to the results, the measured adsorption was not very high, but overall, it was higher on PET MP than on PE and varied depending on the compound, which is in line with our findings from the adsorption isotherms. On the other hand, it has been observed that MPs with a smaller particle size adsorb more pollutants, which seems to be related to a higher surface area. A higher surface area facilitates adsorption by providing more active binding sites on MPs. As can be seen in Figure 3, a reduction in particle size in both MPs increases the adsorption of all macrolide antibiotics as consequence of the increment of the surface area. For instance, considering RXM, results showed that a particle size reduction from 3–5 mm to 300 µm in PET increased its adsorption from 15.5 to 20.6%. Likewise, a decrease in the particle size of PE from 5 mm to 300 µm increased the adsorption of this antibiotic from 2.6% to 17.9%. In PE, this increase of adsorption is more pronounced than in PET, leading to an 11.6% increase in the average adsorption of all macrolides on PE, compared to only 5.1% on PET. In a similar study, Cormier et al. (2022) concluded that there is a tendency indicating higher sorption efficiencies for small particles compared to larger particles in the adsorption of perfluorooctanesulfonic acid on PE [35].

MP dosage is also a significant factor that affects sorption capacity, and it was evaluated. An increasing number of MPs resulted in an increase in the adsorption of the antibiotics by both MPs (Figure S4). As observed, CLM showed an adsorption of 2.51% on PE at a dose of 10 g/L, while it reaches 23.2% at the highest dose (80 g/L). Similarly, RXM showed an adsorption of 9.82% on PET particles at low doses, whereas at high doses, the adsorption increased to a value of 27.8%. A higher dosage of MPs allows for higher adsorption of contaminants to the MPs due to the increased surface of the MPs. These results are in concordance with those reported by Mejías et al. (2023), who observed that increasing the number of MP particles facilitated the adsorption of perfluoroalkyl substances on polyamide (PA) MPs [36].

### 2.4. Influence of Environmental Factors

**Figure 4:**
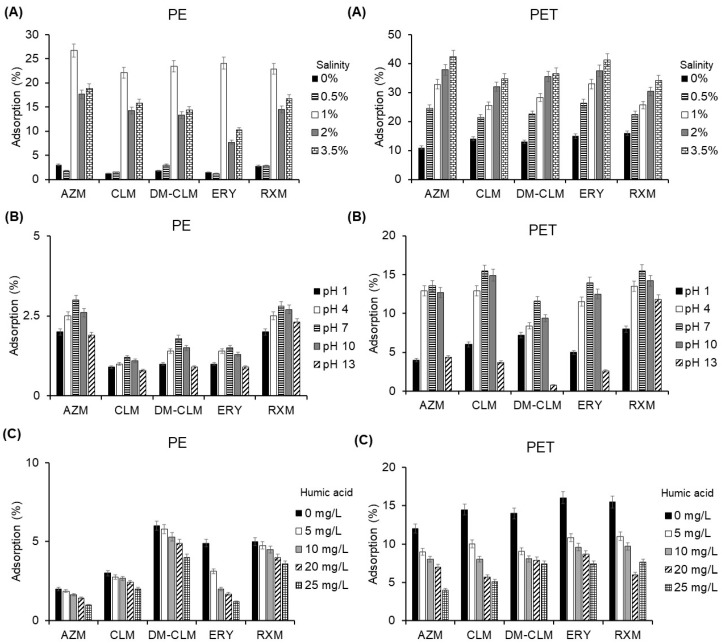
Adsorption of macrolides to PE (**left**) and PET (**right**) in the evaluation of the effect of (**A**) salinity, (**B**) pH, and (**C**) dissolved organic matter.

The factors that most influence the adsorption of antibiotics on MPs were evaluated: salinity, pH, and dissolved organic matter (Figure 4). Salinity was studied to simulate the salinity in aquatic environments, such as rivers, lakes, and oceans. In general, the evaluation of influence of salinity showed different results between the adsorption of the studied macrolides on PE and PET (Figure 4A).

In general, an increase in salinity led to an increase in adsorption percentage of the studied macrolides; however, the compounds exhibited different adsorption behavior between the two MPs (Figure 4A). These results indicate that salinity enhances sorption on PE with a maximum effect observed at a salinity level of 1%. A lower percentage of salinity seems to have only a slight effect, while a higher percentage of salinity results in a reduction of the adsorption of macrolide antibiotics compared to the 1% condition. On the other hand, salinity improved adsorption of macrolides to PET, but in this case increased as the percentage of salinity was higher. Salinity has demonstrated varying influences on the adsorption of organic pollutants to MPs. Some authors have reported that salinity does not follow a consistent tendency when influencing the sorption capacity of these pollutants on MPs, while others have concluded that salinity has a negative impact on adsorption [36,37]. Conversely, some studies have reported that salinity increases sorption of organic compounds, enhancing their water solubility [38]. In this study, salinity had a positive impact on the adsorption of macrolides onto PET, whereas this effect was not observed with PE, which showed higher adsorption at 1% salinity. 

pH is another factor that influences the adsorption of antibiotics on MPs. Environmental water samples exhibit a diverse range of pH, determining the charge of both pollutants and MPs, thereby impacting the adsorption behavior. The effect of pH was evaluated through adsorption experiments and calculating the pH point of zero charge (pH_PZC_) for PE and PET which determinates their charge. The adsorption efficiency of macrolides is maximal at neutral pH, and the adsorption decreases with further alkalinity or acidity of the medium for both MPs (Figure 4B).

The pH_PZC_ values for PE and PET are 4.3 and 4, respectively (Figure S5). When the solution pH exceeds the pH_PZC_, the MP surface becomes negatively charged, attracting positively charged pollutants through electrostatic interaction. Conversely, when the solution pH is below the pH_PZC_, the MP surface becomes positively charged, attracting negatively charged pollutants. On the other hand, macrolides are rather basic compounds, which means that they are protonated at acidic pH levels, but also at moderate basic pH levels. Therefore, in the pH range between the pH_PZC_ value and the p*K*_a_ values of macrolides, MPs are negatively charged, while antibiotics are positively charged. This is the pH range where a major sorption capacity is expected. Based on these premises, the pH of highest adsorption must be in the range from 4 to 8.5, which is in concordance with experimental data. Similarly, Chen et al. (2021) observed that the adsorption amount of tetracycline (with p*K*_a1_, p*K*_a2_ and p*K*_a3_ values of 3.3, 7.7 and 9.7, respectively) to PE increased with the solution pH in acidic conditions and decreased when pH value exceeded 8 [39]. 

Dissolved organic matter has a ubiquitous presence in aquatic ecosystems has been shown to be key in adsorption processes. Organic matter particles, such as humic or fulvic acids, can compete with the active sites of MPs for the binding of pollutants, affecting the adsorption, resulting in a lower adsorption to MPs surface. Therefore, different concentrations of humic acid were evaluated (Figure 4C). A higher content of dissolved organic matter resulted in a lower adsorption of macrolides onto MPs. For example, an increase of dissolved organic matter to 25 mg/L caused a decrease AZM adsorption from 11.3% to 4% on PET particles. Similarly, AZM adsorption TO PE was reduced from 12% to 5.1% at the higher centration of dissolved organic matter. Dissolved organic matter probably decreases adsorption to MPs through competition with contaminants to bind to sorption sites and complexation with the hydrophobic parts of humic and fulvic acids. These findings are in line with those reported by Lara et al. (2021), which found that endocrine-disrupting compounds decrease their adsorption capacity to PA MPs in the presence of dissolved organic matter [40].

### 2.5. Adsorption in Environmental Matrices: Wastewater, Surface Water, and Tap Water

**Figure 5:**
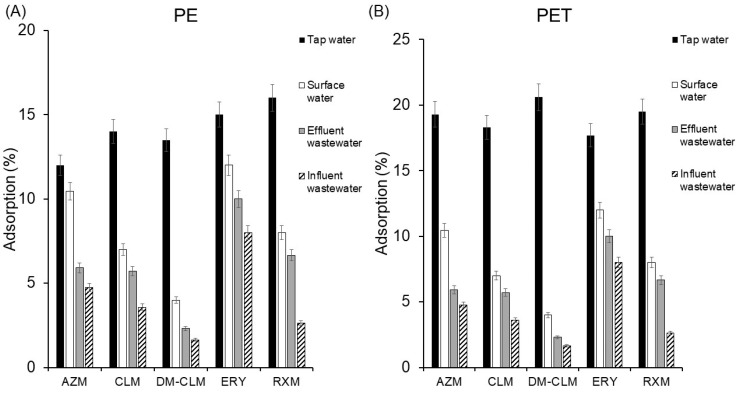
Adsorption of macrolides on PET (**A**) and PE (**B**) in tap water, surface water, and effluent and influent wastewater.

Real environmental aqueous samples are a highly complex matrix that possesses dissolved compounds which could influence the adsorption of macrolides in these matrices. For this reason, even when environmental parameters affecting the adsorption of macrolides on MPs have been studied individually, studies in real environmental aqueous matrices are needed. To this end, antibiotic adsorption was evaluated in tap water, surface water, and effluent and influent wastewater samples under the general conditions. All samples were collected in Seville (southern Spain) during September 2023. Surface water samples were collected from the Guadalquivir River, tap water samples were taken from the laboratory, and wastewater samples were received from a wastewater treatment plant located in Seville. The physicochemical characteristics of the samples are listed in Table S4. It is worth noting that the surface water exhibited high conductivity and ion load. The adsorption results of the studied macrolides on PE and PET in the different aqueous environments are given in Figure 5.

As can be seen, results show that the complexity of the matrix highly reduces the sorption of pollutants to MPs, which correlates with the presence of dissolved organic matter in the four different matrices. Thus, with increasing complexity (tap water < surface water < effluent < influent), a decrease in adsorption percentage is observed. In the case of PE, the increase in matrix complexity resulted in an average decrease in macrolide adsorption from 14.1% in tap water to 4.1% in influent wastewater. Likewise, in PET, the average adsorption decreased from 17.7% in tap water to 4.51% in influent wastewater. The compound most significantly affected in terms of adsorption on PE was RXM, experiencing a decrease in adsorption, by 13%. Meanwhile, DM-CLM was the most adversely influenced in its adsorption on PET, experiencing the highest decrease in adsorption, by 12.5%.

Additionally, a correlation analysis was performed to identify sample characteristics that most affect the sorption behavior on MPs. As can be inferred from Tables S5 and S6, the parameters that most negatively affect adsorption in both types of MPs are pH, Cl^−^, chemical oxygen demand (COD), and total nitrogen (NT). 

COD is a measure of the estimated oxygen required for the oxidation portion of organic matter. It is widely used as an indicator of water quality to determine the amount of biologically active and inactive organic matter. In addition, dissolved organic matter is often predominant in the form of organic C, N, and P [41]. As expected, there is a strong linear correlation between COD, NT, and PO_4_^3−^ due to their relationship with the dissolved organic matter in water. The correlation between COD (mg/L) and the adsorption of studied antibiotics is <0 in all cases, which indicates that they are negatively correlated. More COD (mg/L) is associated with lower drug adsorption to PE. ERY is the antibiotic most strongly affected (correlation coefficient −0.86) by the presence of organic matter, and RXM the least (−0.10). The same behavior was observed for the adsorption of antibiotics to PET. In this case, COD negatively affected to a similar degree all five macrolides. This aligns with the findings found in real samples (Figure 5) and the assessment of the presence of humic acid (Figure 4C). On the other hand, pH and Cl^−^ also showed a strong negative correlation with the adsorption of virtually all studied macrolides, except for RXM in PE particles. 

Similar studies examining other classes of antibiotics on MPs have reported analogous findings. For example, Arvaniti et al. (2022) concluded that the adsorption of two antihypertensive drugs, losartan and valsartan, to PS-MPs exhibited a decrease with increasing matrix complexity, aligning with the results observed in this study [42]. In another study, Anbarani et al. (2023) observed that distilled water exhibited the highest adsorption of tetracycline onto polyvinyl chloride MPs, while municipal wastewater showed the lowest adsorption onto polyvinyl chloride MPs. They attributed this difference to the higher concentration of ions present in municipal wastewater [43].

In Table 2 the main characteristics of some studies about the adsorption of antibiotics onto MPs can be seen. Tetracyclines, sulfonamides, and fluoroquinolones are the most studied groups of antibiotics. Maximum adsorption capacity was in the range from 0.046 to 2.55 mg/g, which is similar to the *q*_max_ reported in this study. The main adsorption mechanisms were hydrophobic interactions, electrostatic interactions, hydrogen bonds, and π–π conjugation. There is no uniformity in the isotherm model; some of them were adjusted to Linear, others to Langmuir, and others to Freundlich models. The concentration tested range was from 0.001 to 50 mg/L and MP size from 1 to 500 μm. In terms of variables tested, we could find that there was a lack of information about the application in real environmental matrices.

## 3. Materials and Methods

### 3.1. Chemicals and Reagents

High-purity antibiotic standard RXM (≥98.0%) was supplied by Sigma-Aldrich (Steinheim, Germany). DM-CLM (≥96.0%) was purchased from Toronto Research Chemicals (Toronto, ON, Canada). CLM ≥ 98.0% and ERY ≥ 98.0% were obtained from Tokyo Chemicals Industry (Eschborn, Germany). AZM (>99.0%) was purchased from European Pharmacopoeia Reference Standard (Strasbourg, France). 

The physical–chemical properties of the target compounds, including their structures, p*K*_a_, and log *K*_ow_ values, are presented in Table S3 in the Supplementary Materials. 

Humic acid and ammonium formate were provided by Sigma-Aldrich (Madrid, Spain). The different forms of MPs (PE powder with a particle size of 300 µm, PE pellets with particle sizes of 2 mm and 5 mm, PET powder with a particle size of 300 µm, and PET pellets with particle sizes ranging from 3–5 mm) were supplied by Goodfellow (Hamburg, Germany). Formic acid, hydrochloric acid, sodium hydroxide, and sodium chloride were provided by Panreac (Barcelona, Spain). LC-MS-grade water and methanol were supplied by Merck (Darmstadt, Germany). Stock solutions of individual standards were prepared at 1000 mg/L in methanol. A mixed solution of all standards was prepared by diluting the previous individual standard solutions in water. In order to avoid co-solvent effects, the amount of methanol in the final solution was the lowest needed.

### 3.2. Microplastic Characterization

MP particles were characterized through different techniques suitable for material characterization, according to the literature [47,48,49]. SEM was performed using an FEI-TENEO scanning electron microscope (FEI, USA). FTIR was conducted with a Cary 630 FT-IR (Agilent, Santa Clara, CA, USA) with attenuated total reflection (ATR) using a spectral range between 4000 and 650 cm^−1^ and a spectral resolution of 4 cm^−1^. XRD was applied to measure crystalline composition using a Bruker D8 Advance A25 diffractometer (Bruker, Ettlingen, Germany) with a Cu-Kα as a radiation source operating at 40 kV and 30 mA. Diffractograms were obtained in a range of 1–70° of 2θ with a step size of 0.03° and a step time of 0.1 s. MPs’ surface areas were measured from their Kr adsorption–desorption isotherms according to the Brunauer–Emmett–Teller (BET) equation using an accelerated surface area and porosimetry system (ASAP 2420) (Micromeritics Instrument, Gloucestershire, UK). The pH_PZC_ was calculated by the drift method using a pHmeter BASIC 20 (Crison Instruments, Barcelona, Spain). Particle size was determined using a Mastersizer 2000 (Malvern, UK) by laser diffraction.

### 3.3. Batch Experiments

Batch experiments were conducted changing the variable to be evaluated and keeping all other variables constant. The standard conditions were 0.5 g of MPs and 30 mL of deionized water containing 0.5 mg/L of contaminants under agitation for 24 h. All experiments were carried out at room temperature and in triplicate. The solutions were agitated in the vials by magnetic stirring at 200 rpm. To account for potential analyte losses due to degradation, procedural blanks without MPs were processed. After each assay and prior to analysis, the aqueous solution was filtered through a 0.22 µm filter. First, adsorption isotherm experiments were performed with varying concentrations of the studied analytes (from 0.1 to 1.5 mg/L), and adsorption kinetics experiments were carried out for 1 min to 24 h. Second, the characteristics of the MPs influencing adsorption capacity were evaluated by assessing different doses (10, 20, 40, 60 and 80 g/L of MPs) and particle sizes (3–5 mm and 300 µm for PET; 5 mm, 2 mm and 300 μm for PE). Third, the impact of environmental conditions on adsorption was investigated by examining variations in pH (1, 4, 7, 10, and 13), salinity (0, 0.5, 1, 2, and 3.5%) and dissolved organic matter (0, 5, 10, 20, and 25 mg/L of humic acid). Lastly, experiments were performed using fortified real aqueous samples at a concentration of 0.5 g/L to simulate real-life scenarios, including tap water, surface water, wastewater influent, and wastewater effluent.

### 3.4. Instrumental and Data Analysis

The analyses of the target antibiotics were performed using high-resolution liquid chromatography–tandem mass spectrometry (LC-MS/MS) applying chromatographic conditions previously reported by Mejías et al. (2022) [50]. A summary of the methodology and LC-MS/MS parameters is provided in Table S7 (Supplementary Materials).

The obtained data were analyzed by applying lineal, Langmuir and Freundlich isotherms models and pseudo-first order and pseudo-second order kinetic models. The corresponding equations and parameter definitions are described in Table S8. Correlation analysis was carried out utilizing Statistica v10.0.

## 4. Conclusions

The adsorption behavior of four macrolide antibiotics and one metabolite was investigated through a series of batch experiments. Results suggested that adsorption isotherms were better fitted to a linear model (*R*^2^ > 0.936 for PE and *R*^2^ > 0.910 for PET) and followed the order PET > PE MPs. All the macrolides studied showed similar adsorption capacities, although these varied according to the type of MP and the environmental conditions. For both MPs, adsorption could involve pore filling, while hydrophobic interactions could be the main adsorption mechanism for PE and hydrogen bonding for PET. MP characteristics significantly influence the adsorption capacity of macrolides. In this attempt, smaller-particle MPs and higher doses of MPs facilitated the sorption of antibiotics. In both cases, this could be attributed to the increased number of active adsorption sites for the organic compounds. Environmental factors possessed a high effect on adsorption capacity. Dissolved organic matter decreased sorption by competition of active sorption binding sites. It has been demonstrated that both PE and PET MPs can serve as a vector of macrolide antibiotics in aquatic environments such as tap water, surface water, and wastewater. In addition, an increase in complexity of the aqueous matrix showed a decrease to the adsorption of these antibiotics. 

These results highlight the complexity of macrolide adsorption onto MPs, which is strongly influenced by environmental factors, MP characteristics (such as polarity, size, shape, etc.), and the complex dynamics of aquatic environments. Consequently, it is essential to investigate the behavior of antibiotics and other emerging contaminants on different types of MPs, and to evaluate their release into the environment. Understanding the impact of MPs contamination is crucial, as MPs serve as carriers and reservoirs of pollutants, affecting their distribution, bioavailability, biomagnification, and toxicity in the aquatic environment.

## Figures and Tables

**Table 1:** Experimental parameters of adsorption isotherms of macrolides and metabolite onto PE and PET.

Model	Parameter	AZM	CLM	DM-CLM	ERY	RXM
**PE**						
Linear	*K*_d_ (L/g)	0.003	0.002	0.008	0.007	0.008
	*R* ^2^	0.936	0.949	0.957	0.957	0.965
Langmuir	*q*_max_ (mg/g)	0.036	0.027	0.067	0.047	0.075
	*K*_L_ (L/mg)	0.306	0.108	0.276	0.394	0.036
	*R* ^2^	0.934	0.919	0.931	0.836	0.936
Freundlich	*K*_F_ (L/g)	0.008	0.007	0.008	0.084	0.019
	*n*	1.773	1.622	1.104	1.642	1.743
	*R* ^2^	0.803	0.940	0.913	0.833	0.748
**PET**						
Linear	*K*_d_ (L/g)	0.007	0.005	0.011	0.009	0.010
	*R* ^2^	0.950	0.988	0.976	0.910	0.966
Langmuir	*q*_max_ (mg/g)	0.714	0.654	1.131	0.875	0.903
	*K*_L_ (L/mg)	0.228	0.005	0.272	0.284	0.016
	*R* ^2^	0.792	0.696	0.957	0.759	0.795
Freundlich	*K*_F_ (L/g)	0.042	0.003	0.007	0.073	0.025
	*n*	1.475	1.852	1.084	1.359	1.595
	*R* ^2^	0.717	0.813	0.953	0.845	0.689

**Table 2:** Studies of antibiotics adsorption onto MPs.

MP Type	MP Size (μm)	Antibiotics	Variables Tested	MPs (mg)/ Water (mL)	Concentration Range (mg/L)	Isotherm Model	*q*_max_ (mg/g)	Mechanism	Reference
PE	150–425	Tetracycline, chlortetracycline, oxytetracycline	pH and salinity	250/30	0–50	Freundlich	0.053–0.064	Non-bond interactions	[39]
PE	45–48	Sulfamethoxazole	-	8/40	0.001–0.1	Linear and Langmuir	0.04609, 0.06438 and 0.0888	Hydrophobic and electrostatic interactions	[34]
PE, PS	25	Norfloxacin	Temperature, pH, dissolved organic matter, heavy metal ions, and salinity	5000/50	5–30	Langmuir	0.231–0.924	π–π conjugation, intermolecular hydrogen bonds, ion exchange, and electrostatic interactions	[44]
PS	1	Tylosin, sulfamethazine, erythromycin	Aging	25/20	1–25	Linear	0.1937–1.161	Hydrogen-bond interactions	[25]
Polylactic acid, PE	100	Ciprofloxacin, norfloxacin	Aging, temperature, pH, dissolved organic matter, salinity, desorption	200/40	1–5	Langmuir	0.2838–2.586	Hydrogen bonding, π–π conjugation, ion exchange, and electrostatic interactions	[45]
PS, PET	62–106	Tetracycline	Aging, Cu, pH, desorption, temperature	100/20	0.5–40	Freundlich	0.192–0.516	Physical interactions	[32]
Polypropylene	500	Sulfathiazole, sulfamerazine, sulfamethazine, sulfamethoxazole, ciprofloxacin, enrofloxacin, ofloxacin, norfloxacin, tetracycline, chloramphenicol	Aging	100/200	5–40	Langmuir	0.33–2.55	Hydrophobic, hydrogen, and electrostatic interactions	[46]
PE, PET	5000–300	AZM, CLM, DM-CLM, RXM, ERY	pH, salinity, dissolved organic matter, size, dosage, real samples	500/30	0.1–1.5	Linear	0.027–1.131	Hydrogen-bond interactions, pore filling, and hydrophobic interactions	This work

## Data Availability

All data generated for this study are contained within the article.

## Supplementary Materials

The following supporting information can be downloaded at: https://www.mdpi.com/article/10.3390/antibiotics13050408/s1, Table S1: Surface area and particle size of PE and PET particles before and after adsorption process; Table S2: Adsorption kinetic parameters; Table S3: Physical-chemical properties of the target compounds; Table S4: Water sources characterization; Table S5: Matrix correlation of macrolides and metabolite adsorption onto PE MPs with water sources physical-chemical characteristics; Table S6: Matrix correlation of macrolides and metabolite adsorption onto PET MPs with water sources physical-chemical characteristics; Table S7: LC-MS/MS parameters; Table S8: Adsorption isotherms and kinetics models studied; Figure S1: FTIR analysis of PE before (upper) and after (down) the adsorption process; Figure S2: FTIR analysis of PET before (upper) and after (down) the adsorption process; Figure S3: XRD analysis for PE before and after the adsorption process (upper) and for PET before and after the adsorption process (down); Figure S4: Adsorption percentage (%) of macrolides and metabolite on PE (upper) and PET (down) MPs in the evaluation of microplastic dosage; Figure S5: Final pH with PE after 48 h of agitation versus initial pH without PE (upper) and final pH with PET after 48 h of agitation versus initial pH without PET (down). Refs. [51,52] are cited in Supplementary Materials.
