# Supplementary material for: Adsorption of Macrolide Antibiotics and a Metabolite onto Polyethylene Terephthalate and Polyethylene Microplastics in Aquatic Environments
Source: Antibiotics (Basel). 2024 Apr 29;13(5):408. doi: 10.3390/antibiotics13050408 (PMC11117229; doi:10.3390/antibiotics13050408)
Supplement: Supplementary file 1 [file antibiotics-13-00408-s001.zip › antibiotics-2973784-SI.pdf]

Supplementary material

Adsorption of Macrolide Antibiotics and a Metabolite onto Polyethylene Terephthalate and Polyethylene Microplastics in Aquatic Environments

Carmen Mejías, Julia Martín *, Laura Martín-Pozo, Juan Luis Santos, Irene Aparicio and Esteban Alonso

Departamento de Química Analítica, Escuela Politécnica Superior, Universidad de Sevilla, E-41011 Seville, Spain; cmpadilla@us.es (C.M.); lpozo@us.es (L.M.-P.); jlsantos@us.es (J.L.S.); iaparicio@us.es (I.A.); ealonso@us.es (E.A.)

* Correspondence: jbueno@us.es; Tel.: +34-954-556-250

Table S1. Surface area and particle size of PE and PET particles before and after adsorption process.

	PE particles 300 μm before adsorption	PE particles 300 μm after adsorption	PET particles 300 μm before adsorption	PET particles 300 μm after adsorption
BET surface area (m^2/g)	0.0686	0.0726	0.1345	0.1444
Single point surface area (m^2/g)	0.0555	0.0601	0.1137	0.1213
Particle size (μm)	252.630	242.985	118.847	125.620

Table S2. Adsorption kinetic parameters.

Compound	PFO model			PSO model		
	R^2	q_e (mg/g)	k_1	R^2	k_2	q_e (mg/g)
**PE**						
CLM	0.661	0.001	0.001	0.968	0.004	0.001
DM-CLM	0.760	0.002	0.002	0.979	0.005	0.002
ERY	0.868	0.001	0.001	0.983	0.004	0.001
RXM	0.756	0.001	0.001	0.987	0.004	0.001
AZM	0.662	0.001	0.001	0.976	0.004	0.001
**PET**						
CLM	0.663	0.004	0.002	0.969	0.006	0.004
DM-CLM	0.667	0.005	0.003	0.977	0.008	0.005
ERY	0.576	0.004	0.002	0.973	0.006	0.004
RXM	0.764	0.004	0.002	0.977	0.006	0.004
AZM	0.865	0.004	0.002	0.984	0.007	0.004

Table S3. Physical-chemical properties of the target compounds.

Compound	Molecular weight (g mol^{-1})	pK_a	Log K_{ow}	Structure
**Roxithromycin (RXM)**	837.0	9.08[a], 12.45[a]	2.75[c]	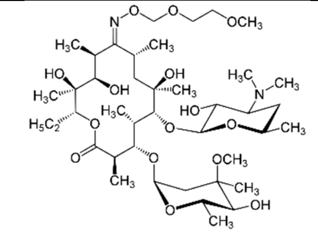
**Azithromycin (AZM)**	749.0	9.57[a], 12.43[a]	4.02[c]	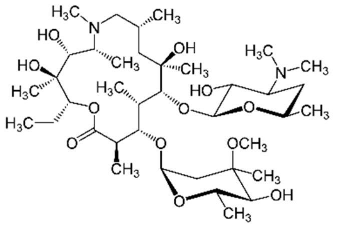
**Erythromycin (ERY)**	733.9	8.38[a], 12.44[a]	3.06[c]	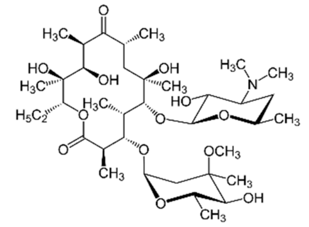
**Clarithromycin (CLM)**	747.9	8.38[a], 12.46[a]	3.16[c]	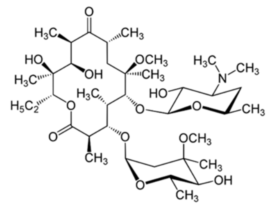
N-desmethylclarithromycin (DM-CLM)	733.9	13.08[b]	-	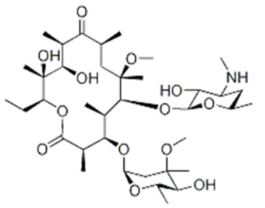

Parent compounds are marked in bold; Abbreviations are written in brackets; -: Not data found. [a]: https://go.drugbank.com/; [b]: https://www.chemicalbook.com/; [c]: Li et al., 2018.

Table S4. Water sources characterisation.

**Water sources**	**pH**	**Cl^- (M)**	**P_T (mg/L)**	**COD (mg/L)**	**N_T (mg/L)**	**σ (mS/cm)**
Tap water	6.59	8.2 E-4	<0.1	22.3	<20	0.44
Surface water	8.15	2.8 E-4	<0.17	31.0	<20	2.07
Effluent wastewater	8.99	2.5 E-3	0.42	32.0	21.1	0.94
Influent wastewater	9.20	2.5 E-3	2.98	171	35.9	1.11

COD: Chemical oxygen demand.

Table S5. Matrix correlation of macrolides and metabolite adsorption onto PE MPs with water sources physical-chemical characteristics.

Variable	pH	Cl^- (M)	PO_4^{3-} (mg/L)	COD (mg/L)	N_T (mg/L)	σ (mS/cm)	AZM Ads (%)	CLM Ads (%)	DM-CLM Ads (%)	ERY Ads (%)	RXM Ads (%)
pH	**1.00**	**0.99**	**0.62**	0.59	0.59	0.37	**-0.95**	**-0.97**	**-0.97**	**-0.89**	0.17
Cl^- (M)		**1.00**	**0.60**	0.57	0.57	0.24	**-0.98**	**-0.98**	**-0.98**	**-0.90**	0.03
PO_4^{3-} (mg/L)			**1.00**	**1.00**	**1.00**	-0.03	**-0.69**	**-0.76**	**-0.75**	**-0.89**	-0.16
COD (mg/L)				**1.00**	**1.00**	0.02	**-0.65**	**-0.73**	**-0.73**	**-0.86**	-0.10
N_T (mg/L)					**1.00**	-0.04	**-0.66**	**-0.73**	**-0.73**	**-0.87**	-0.17
σ (mS/cm)						**1.00**	-0.06	-0.20	-0.20	-0.07	**0.98**
AZM Ads (%)							**1.00**	**0.98**	**0.98**	**0.94**	0.15
CLM Ads (%)								**1.00**	**1.00**	**0.97**	0.01
DM-CLM Ads (%)									**1.00**	**0.97**	0.00
ERY Ads (%)										**1.00**	0.12
RXM Ads (%)											**1.00**

Ads: adsorption; COD: Chemical oxygen demand.

Table S6. Matrix correlation of macrolides and metabolite adsorption onto PET MPs with water sources physical-chemical characteristics.

Variable	pH	Cl^- (M)	PO_4^{3-} (mg/L)	COD (mg/L)	N_T (mg/L)	σ (mS/cm)	AZM Ads (%)	CLM Ads (%)	DM-CLM Ads (%)	ERY Ads (%)	RXM Ads (%)
pH	**1.00**	**0.99**	**0.62**	0.59	0.59	0.37	**-0.92**	**-0.98**	**-0.98**	**-0.97**	**-0,97**
Cl^- (M)		**1.00**	**0.60**	0.57	0.57	0.24	**-0.96**	**-0.95**	**-0.94**	**-0.96**	**-0,94**
PO_4^{3-} (mg/L)			**1.00**	**1.00**	**1.00**	-0.03	**-0.75**	**-0.65**	-0.52	**-0.78**	**-0.75**
COD (mg/L)				**1.00**	**1.00**	0.02	**-0.71**	**-0.64**	-0.51	**-0.76**	**-0.74**
N_T (mg/L)					**1.00**	-0.04	**-0.72**	**-0.62**	-0.49	**-0.76**	**-0,72**
σ (mS/cm)						**1.00**	0.00	-0.50	-0.55	-0.29	-0.41
AZM Ads (%)							**1.00**	**0.87**	**0.82**	**0.95**	**0.90**
CLM Ads (%)								**1.00**	**0.99**	**0.97**	**0.99**
DM-CLM Ads (%)									**1.00**	**0.93**	**0.95**
ERY Ads (%)										**1.00**	**0.99**
RXM Ads (%)											**1.00**

Ads: adsorption; COD: Chemical oxygen demand.

LC-MS/MS determination

Chromatographic determination was performed using an Agilent 1290 Infinity II liquid chromatographic system (Agilent, USA) coupled to a 6495-triple quadrupole (QqQ) mass spectrometer (MS) equipped with an electrospray_ionization source (ESI).

Chromatographic separation was carried out in a Zorbax RRHD Eclipse Plus C18 (150 mm × 3.0 mm i.d., 1.8 µm particle size) column (Agilent, Santa Clara, CA, USA), protected with a Zorbax RRHD Eclipse Plus C18 (3.0 mm i.d., 1.8 µm particle size) guard column (Agilent, Santa Clara, CA, USA) and thermostated at 35 °C. The mobile phase was composed by 10 mM ammonium formate buffer containing 0.05% of formic acid (solvent A) and methanol (solvent B). Elution was carried out at a flow rate of 0.4 mL min^{-1}. Elution started with 5% of solvent B, held 1 min. Solvent B was linearly increased to 30% in 3 min, then to 60% in 8 min and, finally, to 100% in 2 min, held for 2 min. Back to initial conditions was carried out in 2 min and held for 2 min for equilibration. Total run time was 20 min. LC-MS/MS parameters of each compound is presented in Table S7.

The LC system was coupled to a 6410 triple quadrupole mass spectrometer (MS/MS) equipped with an electrospray ionisation source operated in positive mode. The following settings were used: fragmentor, 166 V; capillary voltage, 3000 V; nebuliser pressure, 40 psi; drying gas flow rate, 9 L min^{-1} and gas temperature, 350 °C.

Table S7. LC-MS/MS parameters.

MS/MS parameters								
Compound	Ionization mode	Precursor ion (*m/z*)	Product ions (MRM1/MRM2) (*m/z*)	CE (V)	RT (min)	Ion ratio	LOD (ng/L)	LOQ (ng/L)
CLM	Positive	749.0	158.1/590.4	28/16	18.128	49.5	0.01	0.03
DM-CLM	Positive	734.9	144.1/576.4	144.1/576.4	18.161	23.5	0.02	0.06
ERY	Positive	734.5	83.0/576.4	83.0/576.4	18.140	81.4	0.02	0.06
RXM	Positive	838.1	158.1/679.4	32/20	18.214	72.9	0.01	0.03
AZM	Positive	750.0	591.5/116.1	28/44	18.138	56.1	0.01	0.03

CE: collision energy; RT: retention time; LOD: limit of detection; LOQ: limit of quantification

Data analysis

The amount of adsorbed macrolide (q) was calculated as the difference between its concentration before and after the adsorption experiment according to Eq. (S1):

(S1) $q = (C_i - C_e) \times V/m$

where m is the MPs weight (g), V (L) is the solution volume, C_i (mg/L) and C_e (mg/L) are the concentrations of the macrolides at the beginning and at the end of the adsorption, respectively. Eq. (S2) was used to calculate adsorption percentage (%):

(S2) Adsorption (%) = $(C_i - C_e)/C_i \times 100$

Different mathematical models including Langmuir, Freundlich and Linear were evaluated to determine the adsorbent performance to experimental isotherms. Pseudo-first and pseudo-second order kinetic models were evaluated. Supplementary material, Table S8, shows a summary, including equations and parameter definition.

Table S8. Adsorption isotherms and kinetic models studied.

Isotherm	Model Equation
Langmuir	$q = \frac{q_{max} K_L C_e}{1 + (C_e K_L)}$<br>$C_e$: Equilibrium concentration of pollutants (mg/L);<br>q: Equilibrium adsorption capacity (mg/g);<br>q_{max}: maximum amount adsorbed within a monolayer (mg/g);<br>K_L: Langmuir dissociation constant (L/mg), which is related to the adsorption energy.
Freundlich	$q = K_F C_e^{1/n}$<br>C_e: Equilibrium concentration of pollutants (mg/L);<br>q: Equilibrium adsorption capacity (mg/g);<br>K_F: Freundlich constant (L/mg), which is related to the affinity of the adsorbent to the adsorbate;<br>$1/n$: dimensionless parameter, which indicates how adsorption varies as a function of the concentration.
Linear	$q = K_d \times C_e$<br>C_e: Equilibrium concentration of pollutants (mg/L);<br>q: Equilibrium adsorption capacity (mg/g);<br>K_d: solution-soil distribution coefficient (L/g).
**Kinetic**	**Model equation**
Pseudo-first order (PFO)	$\ln (q_e - q_t) = \ln q_e - k_1 \times t$

	q_e and q_t: amounts of compounds (mg/g) adsorbed at equilibrium and at a t time;<br>t: time (min);<br>k_1: PFO kinetic constants.
Pseudo-second order (PSO)	$t/q_t = 1/(k_2 \text{ x } q_e^2) + t/q_e$<br>$q_e$ and q_t: amounts of compounds (mg/g) adsorbed at equilibrium and at a t time;<br>t: time (min);<br>k_2: PSO kinetic constants.

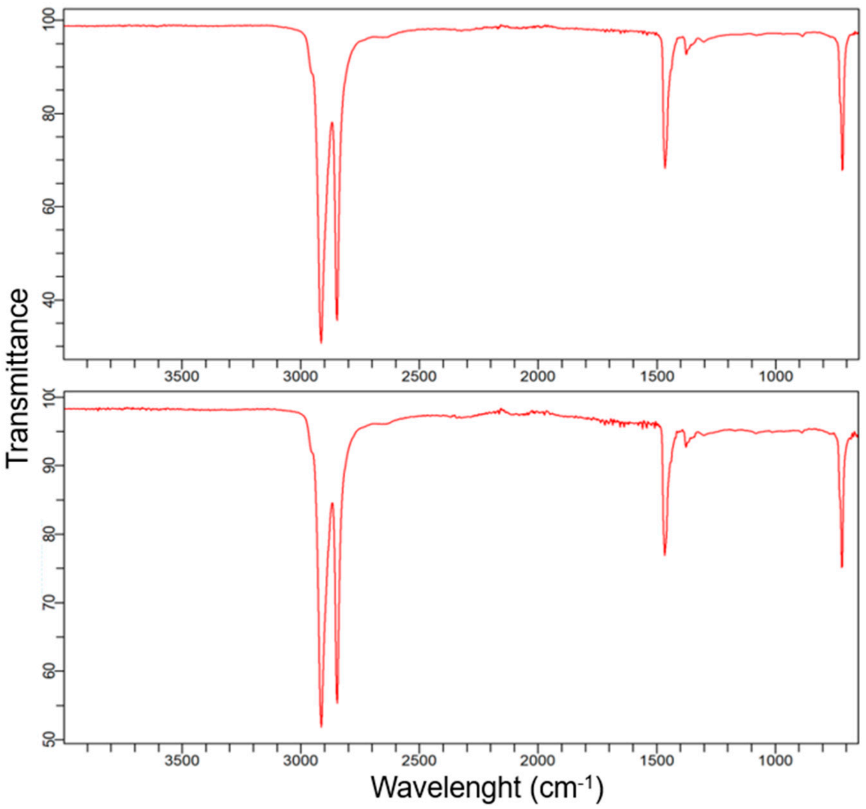

Figure S1. FTIR analysis of PE before (upper) and after (down) the adsorption process.

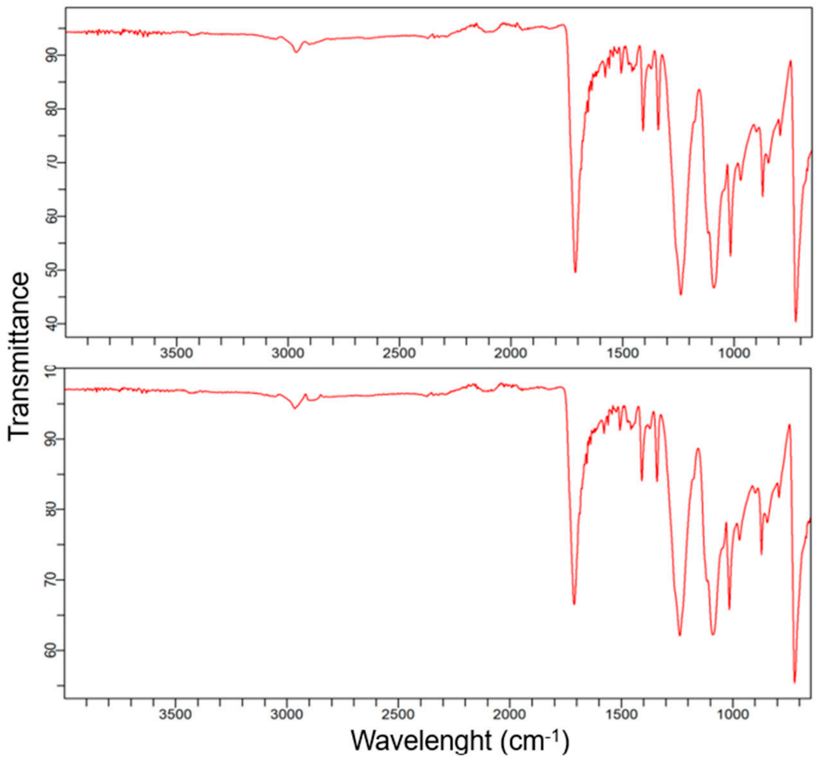

Figure S2. FTIR analysis of PET before (upper) and after (down) the adsorption process.

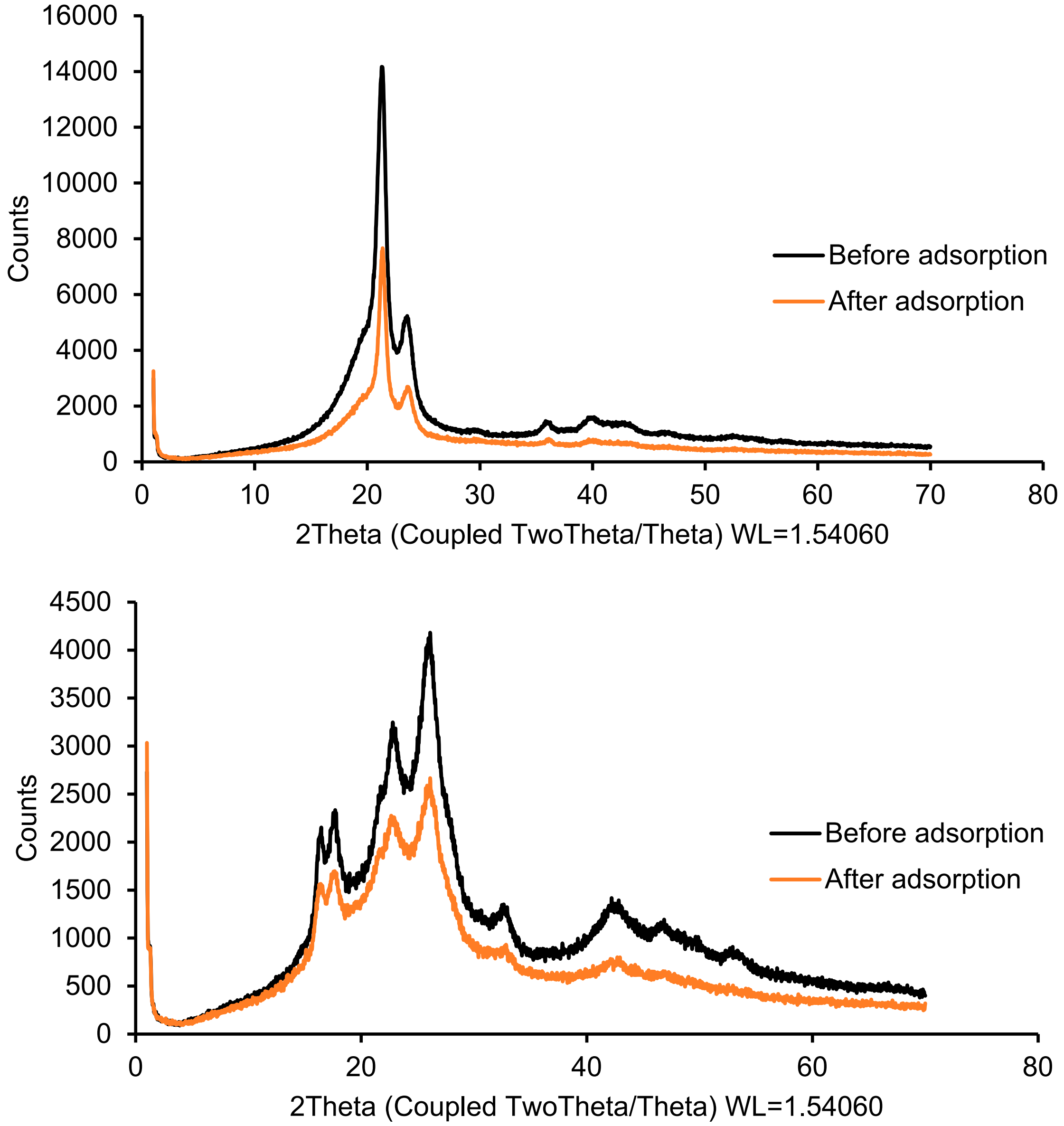

Figure S3. XRD analysis for PE before and after the adsorption process (upper) and for PET before and after the adsorption process (down).

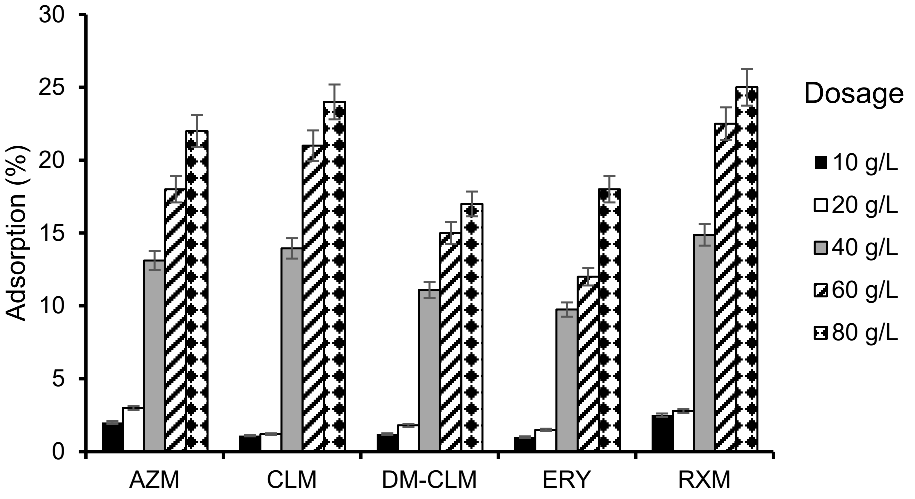

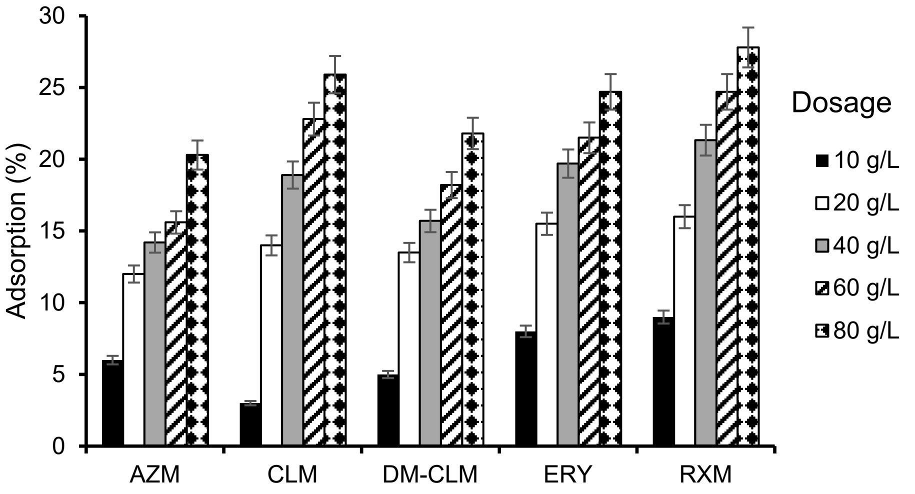

Figure S4. Adsorption percentage (%) of macrolides and metabolite on PE (upper) and PET (down) MPs in the evaluation of microplastic dosage.

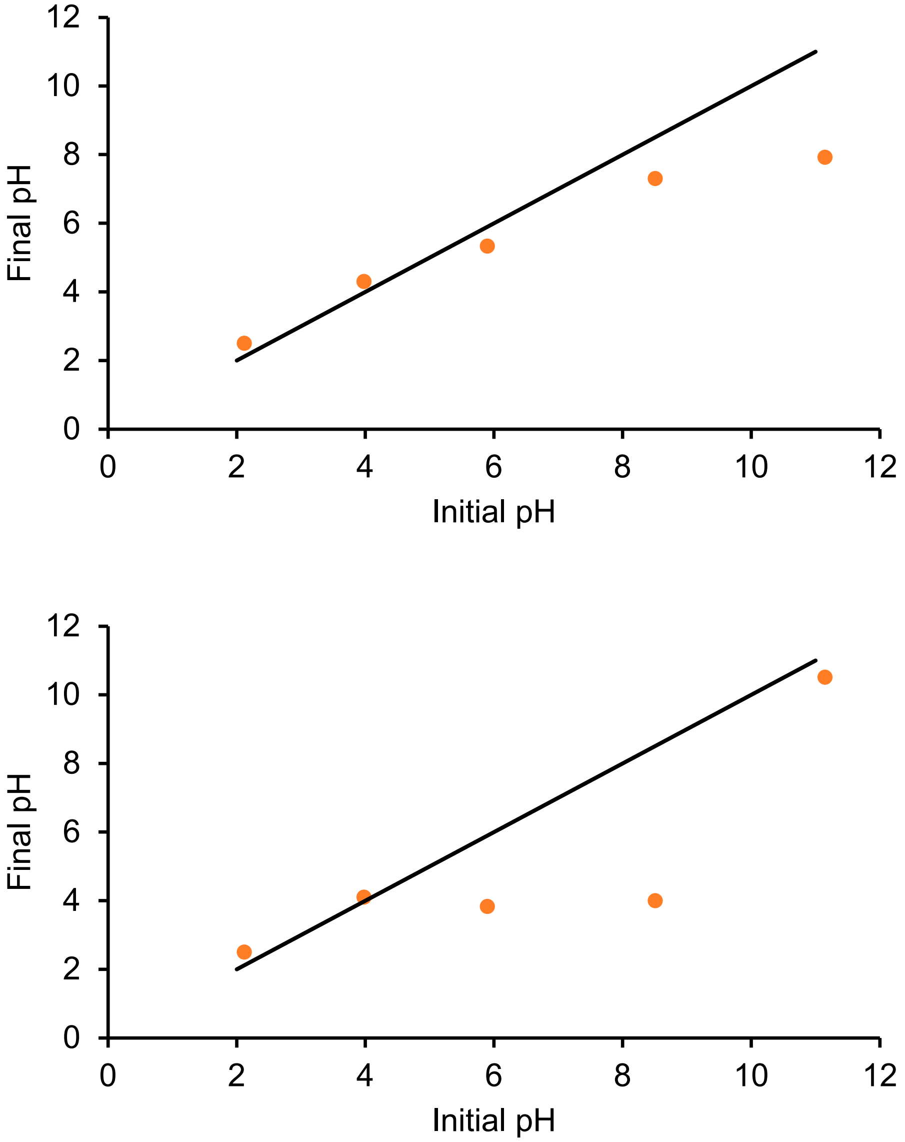

Figure S5. Final pH with PE after 48h of agitation versus initial pH without PE (upper) and final pH with PET after 48h of agitation versus initial pH without PET (down).

Supplementary material references

Chemical BooK. Sourcing and Integrating Center of Chemicals Materials in China. Available on: https://www.chemicalbook.com/

DrugBank Online. Database for Drug and Drug Target Info. Available on: https://go.drugbank.com/
